# Self-Assembly of Soluble Chitosan Derivatives Nanoparticles for Vaccine: Synthesis, Characterization and Evaluation

**DOI:** 10.3390/polym13234097

**Published:** 2021-11-25

**Authors:** Jinbao Liu, Shuang Yu, Wanying Qu, Zheng Jin, Kai Zhao

**Affiliations:** 1 Key Laboratory of Chemical Engineering Process and Technology for High-Efficiency Conversion, College of Chemistry and Material Sciences, Heilongjiang University, Harbin 150080, China; ljb19970107@163.com; 2 Key Laboratory of Microbiology, College of Heilongjiang Province, School of Life Science, Heilongjiang University, Harbin 150080, China; 15504623901@163.com (S.Y.); wanying18845118915@163.com (W.Q.); 3 Institute of Nanobiomaterials and Immunology, School of Life Science, Taizhou University, Taizhou 318000, China

**Keywords:** quaternized chitosan derivative, N-2-HACC, self-assembly, nanoparticle

## Abstract

Herein, a novel chitosan derivative nanoparticle was proposed to function as a delivery carrier. First of all, an improvement was made to the way N-2-hydroxypropyl trimcthyl ammonium chloride chitosan (N-2-HACC) was synthesized. Moreover, the solution to one-step synthesis of N-2-HACC from chitosan (CS) was developed. Different from the previous report, the synthesis process was simplified, and there was a reduction in the amount of 2,3-epoxypropyl trimethyl ammonium chloride (EPTAC) used. With its excellent water solubility maintained, the relatively low degree of substitution was controlled to facilitate the cross-linking reaction. The results obtained from ^1^H-NMR, FTIR spectroscopy, and XRD indicated a smooth EPTAC onto CS for the formation of N-2-HACC with 59.33% the degree of substitution (DS). According to our results, N-2-HACC could be dissolved in various organic solvents, deionized water, 1% acetic acid aqueous solution, and others at room temperature. Finally, a novel chitosan nanoparticle material was prepared using the self-assembly method with β-glycerophosphate sodium (β-GC), with excellent immune properties achieved, thus providing a new strategy for chitosan self-assembled nanoparticles.

## 1. Introduction

Porcine epidemic diarrhea virus (PED) is a pig infectious disease caused by porcine epidemic diarrhea virus (PEDV), causing in particular a large number of death of piglets [1]. PEDV is transmitted by infecting porcine epithelial cells through the fecal–oral route [2]. Vaccine is an important way to prevent this disease [3]. To contain viral transmission, an ideal vaccine is supposed to induce mucosal immune responses. Existing vaccines are ineffective in the control and prevention of PEDV, which makes it necessary to develop high-efficiency vaccines. However, aluminum or mineral oil adjuvants are commonly used as vaccine adjuvants, which have certain toxic and side effects [4]. Polymer nanoparticles, especially nanoparticles of chitosan (CS) and its derivatives, as vaccine adjuvants are the focus of current research [5]. In addition, the nanoparticles of chitosan (CS) and its derivative can induce mucosal immune response, as confirmed in those relevant reports. [6,7].

However, the widespread application of CS has been restricted by its low solubility above pH 6.5 [8]. CS is not soluble in water, dilute alkali solution, or common organic solvents, but only in low-concentration inorganic acid acidic solutions such as dilute acetic acid, dilute hydrochloric acid, and some particular organic solvents. This is attributed to the stereoregularity of its molecular structure and the hydrogen bonding between molecules, which hinders its practical application [9,10,11]. In order to break down the H-bond network of CS and improve the solubility of CS, the introduction of functional groups into CS free amine groups or primary hydroxyl groups for chemical modification has now become the focus of attention for research [12]. The commonly used techniques of modification include carboxymethylation [13], quaternization [12], sulfation [14], and thiolation [15]. These artificially introduced functional groups endow CS with new functional properties, which makes CS and CS derivatives attract much attention for use in various medical and industrial settings [16]. Thus, in order to improve the water solubility of CS, while widening the scope of its application, CS was modified by quaternization and synthesized quaternized chitosan in our previous studies, which is N-2-HACC, thus improving the water solubility of the modified CS derivative significantly [17]. Preparation of CS and CS derivative nanoparticles can be performed by cross-linking polymer chains together with covalent bonds, or by exploiting physical interactions between the polymer chains, such as hydrogen bonds, electrostatic forces, or hydrophobic associations. The attractive forces that can be exploited in the preparation of the particles may lead to both contraction and aggregation of the particles over time. Estimation of the compactness of the nanoparticles, along with their size, can help to discriminate between intra- and interparticle associations [18]. CS is readily soluble in an acidic environment due to protonation of the amine groups. The resultant positive charge makes it possible to prepare nanoparticles by ionotropic gelation with multivalent anions, such as tripolyphosphate (TPP) [19]. The same reaction can be achieved if there is a certain amount of retention of amine groups in CS derivatives, but TPP can be irritating to eyes and skin, which limits its use. We propose to replace TPP with β-glycerophosphate sodium (β-GC). In addition, it is understood that there is no relevant report on the application of N-2-HACC and β-GC to the preparation of nanoparticles in the field of vaccines. However, the introduction of hydroxypropyl trimethyl ammonium chloride group will reduce the number of NH_2_ functional groups on N-2-HACC, thus affecting the effect of crosslinking into nanoparticles. The way in which to ensure good water solubility at a lower degree of substitution is an urgent problem to be solved in the preparation of N-2-HACC.

This study was purposed to develop the N-2-hydroxypropyl trimcthyl ammonium chloride chitosan (N-2-HACC) with a relatively low degree of substitution while ensuring its excellent water solubility as required for the cross-linking reaction. For this reason, each step-in synthesis was optimized to apply control on the mild reaction conditions, reduce the length of reaction, and reduce the amount of EPTAC used and the degree of substitution (DS). Finally, N-2-HACC and β-GC were self-assembled and cross-linked to generate nanoparticles, and the immune function was studied to evaluate its potential of practical application. Nanoparticles prepared by this method only need to be mixed with PEDV according to the proportion, and there is no need to change the production process of PEDV vaccine. In a small amount (1%), it can significantly improve the immune effect of the vaccine and the immune cycle. It is an ideal vaccine adjuvant candidate.

## 2. Materials and Methods

### 2.1. Materials

Chitosan (CS) with the molecular weight of 713 kDa and deacetylation degree of 85% was purchased from Sinopharm Chemical Reagent Co. Ltd. (Shanghai, China), while acetic acid (AR) and β-glycerophosphoric (β-GC) acid disodium salt was provided by Tianli Chemical Reagent Co., Ltd. (Tianjin, China). Sodium hydroxide (NaOH) and isopropanol (IPA) were purchased from Yongda Chemical Reagent Co., Ltd. (Tianjin, China); 2,3-epoxypropyl trimethyl ammonium chloride (EPTAC) was sourced from Dibo Biotechnology Co., Ltd. (Shanghai, China); and anhydrous ethanol was obtained from Fuyu Fine Chemical Co., Ltd. (Tianjin, China).

### 2.2. Synthesis of the N-2-HACC

**Figure 1:**
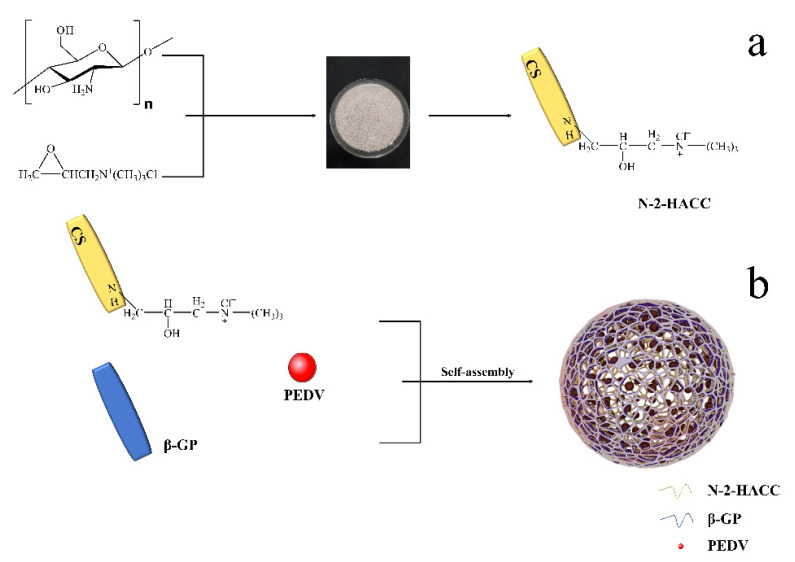
The schemes of synthesis of materials. (**a**) N-2-HACC; (**b**) N-2-HACC/PEDV NPs.

N-2-HACC was synthesized using CS and EPTAC. The synthetic route of N-2-HACC is illustrated in Figure 1. First of all, 6 g of CS was dissolved in acetic acid solution (66.6 mL acetic acid + 173.4 mL deionized water). Secondly, the pH value of the above liquid was adjusted to 9 with 15 mol/L NaOH solution for 0.5 h of soaking. Thirdly, the precipitate was washed to neutral with deionized water, and then dried in a vacuum freeze dryer (SJIA-10N-80C, Ningbo Shuangjia Instrument Co., Ltd., China). Fourthly, the freeze-dried product was dispersed in 50 mL of isopropanol solution, 80 °C water bath, with EPTAC isopropanol solution (9 g/50 mL) added dropwise within 30 min, wherein the reaction was 9 h and 150 mL ice-cold anhydrous ethanol was added; then, the mixture was soaked for 0.5 h, suction filtered, and vacuum freeze-dried to constant weight. The yield (*Y*) was gravimetrically determined using the following equations:

(1) $$W_{2}=\left(W_{1}\times 328.5/162\right)$$

(2) $$Y\left(\%\right)=\left(W_{3}/W_{2}\right)\times 100\%$$

where *W*_1_ (g) represents the added quality of CS, *W*_2_ (g) indicates the theoretical generation quality of N-2-HACC, *W*_3_ (g) denotes the actual generation quality of N-2-HACC, 162 indicates the molecular weight of the CS monomer, and 328.5 refers to the molecular weight of the N-2-HACC monomer.

### 2.3. Characteristics of the N-2-HACC

#### 2.3.1. FTIR and ^1^H NMR Spectroscopy

The structure of N-2-HACC and CS was characterized by Fourier transform infrared (FTIR) spectroscopy (Spectrum one, Perkin-Elmer, Waltham, MA, USA). All samples were conducted with KBr pellets in the range of 400–4000 cm^−1^ at room temperature. The chemical structures of N-2-HACC and CS were confirmed by ^1^H-NMR spectra (Ph = 3) at 25 °C in deuterated acetic acid (CD_3_COOD) (90 pulses and 16 scans) on a Bruker Avance-600 spectrometer (Avance 600 MHz NMR, Bruker, Germany).

#### 2.3.2. X-ray Diffraction

X-ray diffraction (XRD) patterns of CS and N-2-HACC were recorded on an advance X-ray diffractometer (D8 Advance, Bruker AXS, Germany) with Cu-Kα radiation (λ = 0.154 nm), operating at 40 kV and 50 mA. The diffraction data were collected at 2θ values from 5° to 80°, and the scanning rate was 1°/min.

#### 2.3.3. Degree of Deacetylation of CS

At room temperature, 0.2 g of fully dried CS was weighed and added into 16 mL 0.1 mol/L hydrochloric acid (HCl) solution by stirring with the assistance of a magnetic stirrer. After CS was fully dissolved, the solution was diluted to 100 mL with deionized water. Then, the above solution was titrated with 1.000 mol/L NaOH standard solution. Meanwhile, the conductivity (k) at the corresponding volume was recorded, with the conductivity (k) as the ordinate and the NaOH volume (*V*) as the abscissa. The degree of deacetylation (*D.D.*) was calculated using Equation (3):

(3) D.D.=∆V×C×0.001×16/(M×0.0994)

where Δ*V* (L) represents the volume difference of NaOH consumed between the inflection points of the two conductivity changes, *C* (mol/L) indicates the concentration of NaOH, *M* (g) stands for the sample weight, 16 indicates the molecular weight of the amino group, and 0.0994 refers to the theoretical amino content.

#### 2.3.4. Viscosity Measurement

The viscosity was performed in a 0.1 mol/L acetic acid/0.2 mol/L sodium chloride (NaCl) buffer at 25 ± 0.5 °C using a Ubbelohde viscometer. Briefly, 0.3 g of fully dried CS and 0.25 g of fully dried N-2-HACC were weighed, while a 50 mL sample solution was prepared with 0.1 mol/L acetic acid/0.2 mol/L sodium chloride (NaCl) solvent, with the concentration of this solution denoted as C_1_. The solution was filtered using a sand core funnel, and then 10 mL of the filtered solution was added to a tube of a Ubbelohde type viscometer. The temperature of the constant temperature water bath was set to the range of 25 ± 0.5 °C, and the Ubbelohde typed viscometer was vertically fixated in the constant temperature water bath for more than 10 min, so as to balance the temperature of solution with that of the water bath.

The C tube was blocked with one hand, and the solvent was slowly pumped into the G ball of the B tube with the ear wash ball. When pumping was stopped, the C tube was loosened and allowed to pass through the atmosphere, thus making the solution fall naturally inside the capillaries. A stopwatch was used to record the exact time T_1_ between the flow of the solution through the a and b marks. The test was repeated three times, with the average calculated. The interval between two groups was no longer than 0.2 s. The solution was diluted by adding 5 mL, 10 mL, 10 mL, and 10 mL of solvent to tube A in sequence, with the concentrations were recorded as C_2_, C_3_, C_4_, and C_5_, respectively. The outflow time was measured separately and then denoted as T_2_, T_3_, T_4_, and T_5_, respectively.

Prior to the measurement of sample solution, the solvent was measured as described above, with the volume of required solvent set to 10 mL and the measured time denoted as T_0_.

The intrinsic viscosities [*η*] of CS and N-2-HACC solution were determined through the stepwise dilution of viscosity. According to the measured [*η*], Mark–Houwink empirical Equation (4) was applied to calculate viscosity-average molecular weight.

(4) $$\left[\eta \right]=KM_{\eta }^{\alpha }$$

In an adequately diluted polymer solution, *η_sp_*/*C* and ln*η_r_*/*C* and *C* conform to the following Equations (5) and (6):

(5) $$\frac{\eta_{sp}}{C}=\left[\eta \right]+\kappa {\left[\eta \right]}^{2}C$$

(6) $$\frac{\ln\eta_{r}}{C}=\left[\eta \right]-\beta {\left[\eta \right]}^{2}C$$

where *κ* represents Huggins constant, *β* indicates Krameo constant, [*η*] denotes intrinsic viscosity, *η_sp_*/*C* refers to reduced viscosity, *η_sp_* means increased specific viscosity, *η_r_* stands for relative viscosity, *η* indicates viscosity of solution, and *C* (g/L) refers to the concentration of solution.

The method used to obtain [*η*] is shown in Figure S2, where ln*η_r_*/*C* and *η_sp_*/*C* are plotted on the ordinate, while C is plotted on the abscissa to obtain two straight lines. The equations of the two lines are expressed as Equations (5) and (6), respectively. The slopes of the two regression lines, indicated by [*η*]_1_ and [*η*]_2_, respectively, were calculated. The average value of [*η*]_1_ and [*η*]_2_ is the desired [*η*].

#### 2.3.5. Degree of Substitution of N-2-HACC

The degree of substitution of N-2-HACC was to titrate N-2-HACC (2 mg/mL) in 50 mL deionized water with 0.05 mol/L AgNO_3_ at room temperature by conductivity titration. In short, 0.4 g of N-2-HACC was dissolved in deionized water, which was diluted to 100 mL. Then, 25 mL of the solution was pipetted into the beaker, and 25 mL of deionized water was added into the beaker for dilution. Next, it was titrated with 0.05 mol/L AgNO_3_ solution. The degree of substitution (*DS*) was calculated using Equation (7):

(7) $$DS=\frac{V\times C\times 10^{-3}}{V\times C\times 10^{-3}+\left(W-V\times C\times 10^{-3}\times M_{N-2-HACC}\right)/M_{CS}}\times 100\%$$

where *C* (mol/L) represents the concentration of AgNO_3_ solution, *V* (mL) indicates the volume of AgNO_3_ solution, *W* (g) denotes the mass of the titrated N-2-HACC, *M_CS_* means the molecular weight of CS, and *M_N-2-HACC_* refers to the molecular weight of CS substituted by quaternary ammonium salt groups.

#### 2.3.6. Zeta Potential and Size Distribution

The zeta potential of N-2-HACC, and zeta potential and size distribution of N-2-HACC NPs were measured by a laser particle size analyzer (Zeta PALS, Brookhaven, USA). N-2-HACC nanoparticles (N-2-HACC NPs) were dispersed in deionized water at room temperature, and 3 mL 1 mg/mL solution or suspension was placed in the sample tank to measure.

#### 2.3.7. Solubility Test

Solubility test was conducted to determine the solubility of CS and N-2-HACC in different solvents. The solvent was comprised mainly of 0.9% (*w/v*) normal saline, isopropyl alcohol, deionized water, 1% (*w/v*) acetic acid, and 1% (*w/v*) sodium hydroxide.

A total of 1 mL of solvent was measured and added into the centrifuge tube. Then, the sample was added into the centrifuge tube for testing, and vortex oscillation was performed at room temperature until the solution reached saturation. The saturated solution was centrifuged at 4 °C, 15,285× *g*, for 10 min, with the supernatant removed after centrifugation. Then, the insoluble substance was dried and weighed to assess the quality of dissolved sample. Solubility was expressed as the amount (mg) of the test sample dissolved in 1 mL of solvent. Solubility test was performed in triplicate, and the results were analyzed using a statistical method.

### 2.4. Nanoparticle Synthesis and Immune Effect Evaluation

First of all, 2 mL of sorbitan monooleate (span80), 10 mL of petroleum ether, and 20 mL liquid paraffin as the oil phase were added into a beaker. Then, 3 mL of N-2-HACC as the water phase was added into the beaker dropwise and stirred at 900 r/min for 15 min. Next, 1 mL β-GP was added and stirred for 4 min at the same speed, so as to obtain a pre-emulsion, which was then poured into a rapid emulsification device. The pre-emulsion penetrated the membrane (with a pore size of 0.45 μm) 3 times under a nitrogen pressure of 0.15 MPa, before curing in a 37 °C water bath for 3 h. Finally, the solution was centrifuged at 15,285× *g* for 10 min at 4 °C, with the supernatant removed. This was repeated three times for lyophilization.

Diluted to varying concentrations at 0.5%, 1%, 5%, and 10% (*w*/*v*) and mixed with the same amount of PEDV after inactivation, N-2-HACC NPs were stirred at a high speed of 8916× *g* for 30 min to obtain the N-2-HACC/PEDV inactivated vaccine with different doses of N-2-HACC NPs. The vaccine components of different N-2-HACC NPs supplemented doses are shown in Table 1. Care of laboratory animals and experimentation on animals were in accordance with animal ethics guidelines and approved protocols. All the animal studies were approved by the Experimental Animal Ethics Committee of Heilongjiang University (ethic approval number: 20190304001). A total of 25 healthy guinea pigs with negative PEDV serum antibodies were randomly divided into 5 groups. The injection doses are detailed in Table 1 With each group of guinea pigs raised in the same environment, the second booster of immunization was administered in the same way 15 days after the first-time vaccination. After immunization, blood was collected on a weekly basis to detect the antibody titer of ELISA in the serum, and the trend of antibody changes in ELISA was observed to determine the optimal dosage of N-2-HACC NPs. The N-2-HACC solution of the optimal adjuvant dose as determined above was taken for the thorough mixing with the inactivated PEDV, and then stirred at 8916× *g* for 30 min. Finally, the N-2-HACC/PEDV inactivated vaccine was produced.

## 3. Results and Discussion

### 3.1. Characterization of the N-2-HACC

The synthetic route of N-2-HACC is illustrated in Figure 1. N-2-HACC was presented as faint yellow to white powder after freeze-drying, and the yield (Y) of N-2-HACC reached 84.41%.

#### 3.1.1. FTIR and ^1^H NMR Spectroscopy

**Figure 2:**
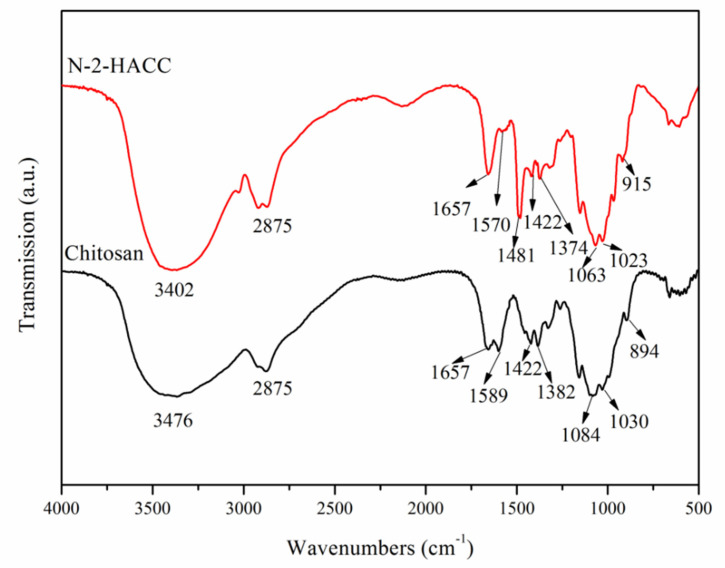
FTIR spectra of CS and N-2-HACC.

FTIR spectra of CS are shown in Figure 2. At 3476 cm^−1^, the peaks of stretching vibration absorption attributed to N–H and O–H were observed [20]. The peak of stretching vibration absorption attributed to saturated C–H (-CH_3_ and -CH_2_) appeared at 2875 cm^−1^, while the bending vibration absorption peak emerged at 1422 cm^−1^ [21]. An absorption peak attributed to the residual acetylamino group (C=O) appeared at 1657 cm^−1^ [22], while a peak of stretching vibration attributed to the amino group (-NH_2_) appeared at 1589 cm^−1^ [23]. An absorption peak attributed to the residual acetylamino group (C-N) appeared at 1382 cm^−1^, and an absorption peak attributed to C–O–C appeared at 1084 cm^−1^. An absorption peak attributed to C–OH appeared at 1030 cm^−1^, while an absorption peak attributed to β-glucosidic bond appeared at 894 cm^−1^ [24].

The FTIR spectrum of N-2-HACC was highly similar to that of CS. Among them, the peak of stretching vibration attributed to the amino group at 1570 cm^−1^ was severely weakened, indicating that the hydrogen atom of the CS amino group was partially substituted with -CH_2_CH(OH)CH_2_N^+^(CH_3_)_3_Cl^−^ [25,26]. In addition, an evident absorption peak of C–H bending vibration attributed to methyl (CH_3_) appeared at 1481 cm^−1^, which validated that methylation had occurred on chitosan [21]. Therefore, N-2-HACC was successfully prepared.

**Figure 3:**
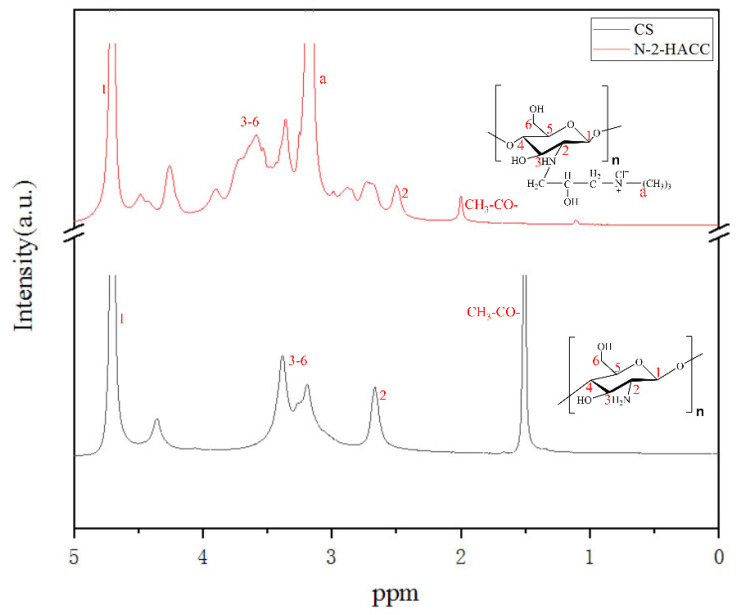
^1^H-NMR spectra of CS and N-2-HACC.

As shown in Figure 3, for the ^1^H-NMR spectrum of CS, the proton signal peak of H1 on the hetero ring appeared at 4.692 ppm, and the proton signal peak of the methyl moieties of the acetyl group appeared at 1.507 ppm [27]. For the ^1^H-NMR spectrum of N-2-HACC, the proton signal peak of H1 on the hetero ring appeared at 4.692 ppm, and the -N^+^(CH_3_)_3_ proton signal peak on the N-2-HACC branched chain appeared at 3.170 ppm [28], indicating the occurrence of substitution reaction on the C-NH_2_ of CS. The FTIR and ^1^H-NMR spectra of CS and N-2-HACC suggest a success in the introduction of EPTAC into the free amino group of CS, which means the smooth generation of target product N-2-HACC.

#### 3.1.2. XRD

**Figure 4:**
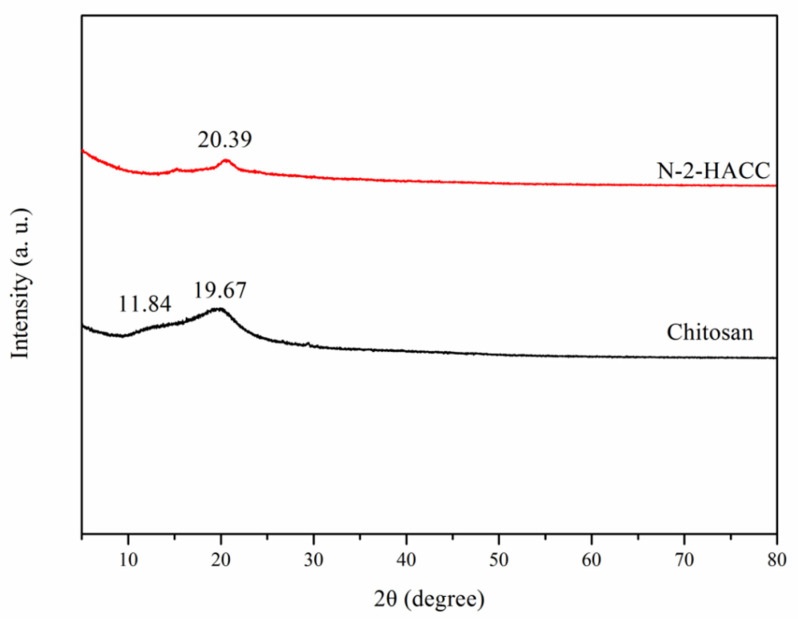
XRD of CS and N-2-HACC.

XRD was applied to explore the crystalline properties of CS and N-2-HACC. According to the XRD curve of CS shown in Figure 4, a clearly visible peak of diffraction absorption appeared at around 19.67°, which is the characteristic peak of a representative crystal structure. At around 11.84°, a smaller peak of diffraction absorption also appeared, which is attributable to the intramolecular and intermolecular hydrogen bonding of the -NH_2_ and -OH groups of CS, indicating the presence of a crystal structure in the CS molecule [29].

According to the XRD curve of N-2-HACC shown in Figure 4, the intensity of diffraction absorption peak was sharply reduced at about 20.39°, while the peak of diffraction absorption was hardly visible at about 11.84°. It is suggested that the introduction of quaternary ammonium salt groups weakened the hydrogen bonding of CS molecules, with the original crystal structure of CS destroyed, thus causing the crystallization peak to vanish [30]. However, water molecules are more likely to be close to such loose amorphous macromolecules, and the hydrophilic groups contained in N-2-HACC synthesized after CS modification can form hydrogen bonds with water molecules again, thus improving the solubility of N-2-HACC and achieving high water solubility.

#### 3.1.3. Degree of Deacetylation of CS

After the dissolution of CS in HCl solution, HCl reacted with -NH_2_ on CS to generate R-NH_3_Cl. The reaction solution was neutralized with a standard NaOH solution. The first neutralizing with excess HCl and neutralizing R-NH_3_Cl after excess HCl was completely neutralized by NaOH. The reaction can be expressed as R-NH_3_Cl + Na^+^ + OH^−^ = R-NH_2_ + Cl^−^ + Na^+^ + H_2_O.

**Figure 5:**
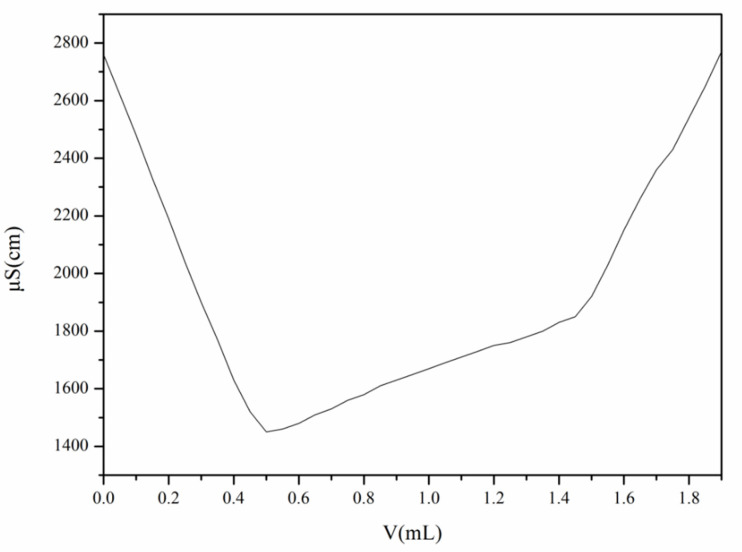
The typical conductometric titration curves of N-2-HACC.

The occurrence of the first turning point resulted from the improvement of conductivity (k) caused by the increase of Na^+^ and Cl^−^. When the NaOH standard solution fully reacted with R-NH_3_Cl, the addition of excess NaOH led to an increase of conductivity (k), thus resulting in the second turning point. The amount of NaOH consumed between the two turning points was equal to that of -NH_2_ in CS. The conductivity (k) was taken as the ordinate, and the volume (V) of NaOH was treated as the abscissa to plot the curve, as shown in Figure 5. As for D.D. of CS, it was calculated as 80.48% using Equation (3).

#### 3.1.4. Viscosity Measurement

The intrinsic viscosity of CS was determined through conventional stepwise dilution. Table 2 lists the experimental data on the intrinsic viscosity of CS.

**Figure 6:**
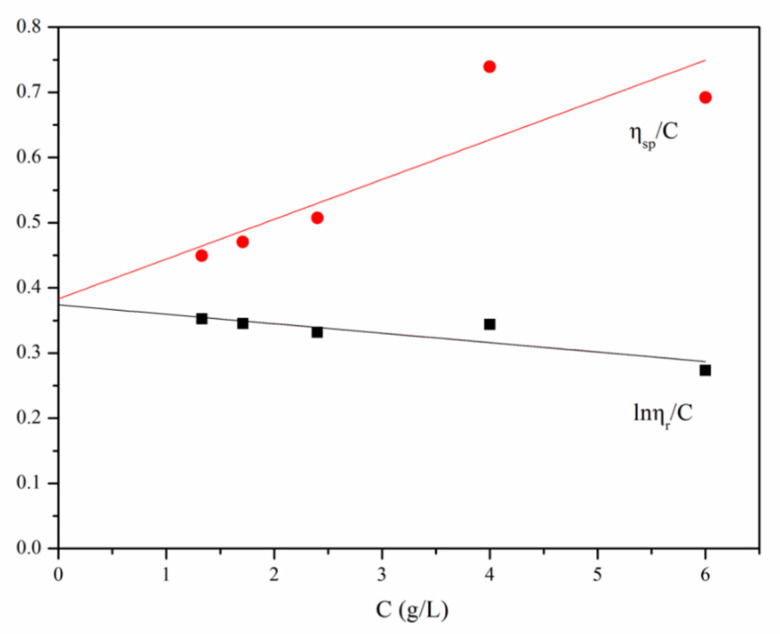
The relationship between *C*-ln*η_r_/C* and *C*-*η_sp_/C* of CS. T = 25.15 ± 0.5 °C.

The curve shown in Figure 6 was plotted according to the experimental data shown in Table 1. The calculation was performed using Equations (5) and (6), [η] = 0.3786 × 10^3^, while the viscosity average molecular weight was calculated using Equation (4), where K = 1.81 × 10^3^, α = 0.93, which led to the result that M_η_ = 5.3 × 10^5^.

The intrinsic viscosity of N-2-HACC was determined by means of conventional stepwise dilution. The experimental data on the intrinsic viscosity of N-2-HACC are shown in Table 3.

**Figure 7:**
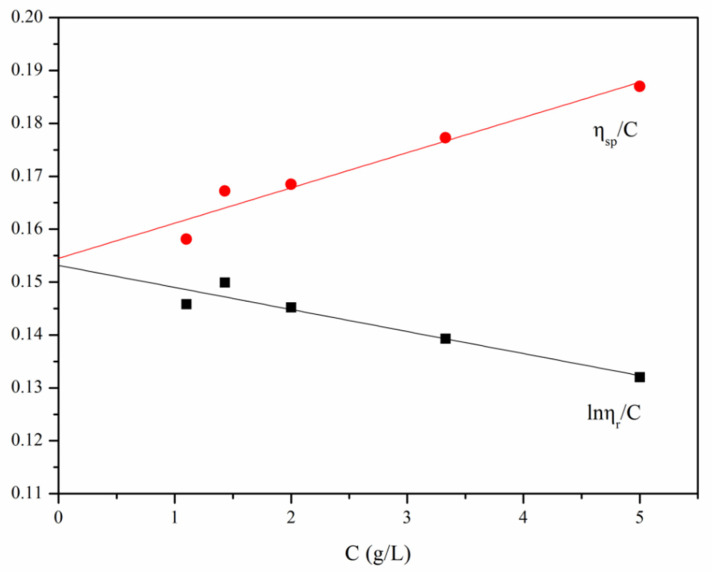
The relationship between *C*-*lnη_r_/C* and *C*-*η_sp_/C* of N-2-HACC. T = 25.15 ± 0.5 °C.

The curve shown in Figure 7 was plotted according to the experimental data listed in Table 2. The calculation was performed according to Equations (5) and (6), [η] = 0.15385 × 10^3^, while the viscosity average molecular weight was calculated using Equation (4), where K = 1.81 × 10^3^, α = 0.93, which led to the result that M_η_ = 2 × 10^5^.

Compared with CS, the viscosity of N-2-HACC was lower than that of CS. The possible reason was that due to the introduction of quaternary amino groups, the hydration of quaternary amino groups and its own strong steric hindrance resulted in a decrease in viscosity. Consistent with previous reports, N-2-HACC has a lower intrinsic viscosity and is an ideal injectable material used in the field of vaccines [31].

#### 3.1.5. Degree of Substitution of N-2-HACC

**Figure 8:**
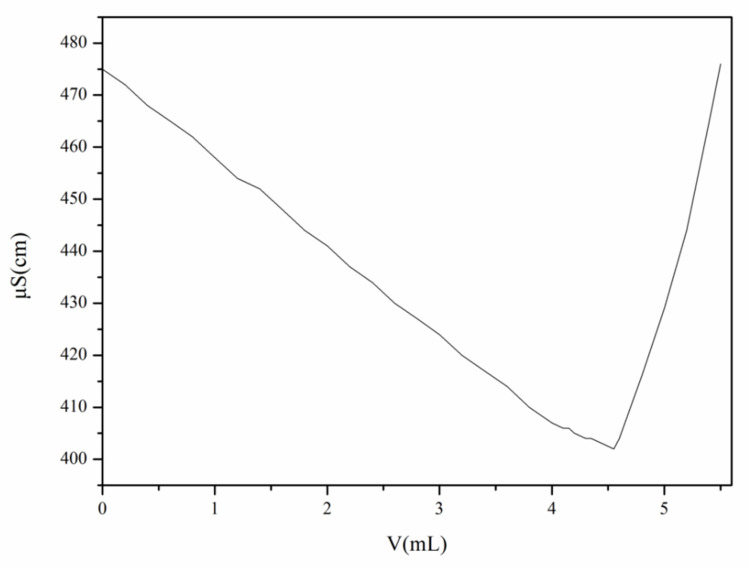
The curve of conductivity of solution with volume of AgNO_3_.

In this study, AgNO_3_ was used as a titrant, and the degree of substitution of N-2-HACC was determined by conductometric titration. According to the principle of Ag^+^ + Cl^−^ → AgCl, the Cl^−^ contained in N-2-HACC solution was titrated with AgNO_3_ solution, with the volume (V) of AgNO_3_ and conductivity (k) recorded. As shown in Figure 8, conductivity (k) also diminished due to the precipitation of AgCl caused by the reaction. When Cl^−^ was completely converted into AgCl, conductivity (k) reached its minimum. Then, with the addition of excessive AgNO_3_, conductivity (k) improved with the increase of Ag^+^ and NO3^−^, which is consistent with previous reports [32]. The curve shown in Figure 8 was plotted with the conductivity (k) as the ordinate and the volume (V) of AgNO_3_ as the abscissa. DS of N-2-HACC was calculated as 59.33% using Equation (7).

#### 3.1.6. Zeta Potential

As shown in Figure S1, the zeta potential of N-2-HACC was assessed using a laser particle size analyzer. Consistent with previous studies, N-2-HACC (zeta potential = +27.55 ± 4.78 mV) possessed higher zeta potential values than the original chitosan particles [33]. The positive charge was attributed to the introduction of positively charged quaternary ammonium groups on the amino groups of chitosan [34]. The positively charged N-2-HACC effectively bound the negatively charged phosphate groups of β-GP through electrostatic attraction to prepare nanoparticles, a vaccine adjuvant, and drug delivery carriers. For biological agents, the ease of positively charged nanoparticles to adhere to the surface of biological mucosa is conducive to the targeted delivery of mucosal drugs. At the same time, the positively charged nanoparticles can encapsulate and adsorb negatively charged nanoparticles or drugs, as required to prepare composite nanoparticles.

#### 3.1.7. Solubility Test

To evaluate the samples for their stability and solubility, we dissolved both CS and N-2-HACC into different solvents, respectively. According to the results shown in Table 4, CS failed to dissolve in normal solvents except 1% (*w/v*) acetic acid aqueous solution (35.1 ± 2.12 mg/mL). By contrast, N-2-HACC exhibited high solubility in 0.9% (*w/v*) normal saline (63.9 ± 0.71 mg/mL), deionized water (73 ± 1.41 mg/mL), 1% (*w/v*) acetic acid aqueous solution (188.9 ± 4.95 mg/mL), and 1% (*w/v*) sodium hydroxide aqueous solution (54.1 ± 0.99 mg/mL).

It can be judged from above that CS can be dissolved only in weak acid solution, rather than in neutral and weak alkaline solutions. However, the N-2-HACC derived from the quaternization of CS can be dissolved in acidic, neutral, and alkaline solutions, suggesting that N-2-HACC possesses high solubility, which is conducive to improving the solubility of CS and widening the scope of application for CS. This is because N-2-HACC has its own soluble quaternary ammonium salts and does not need to be produced only under acidic conditions like chitosan.

### 3.2. Preparation of N-2-HACC NPs

**Figure 9:**
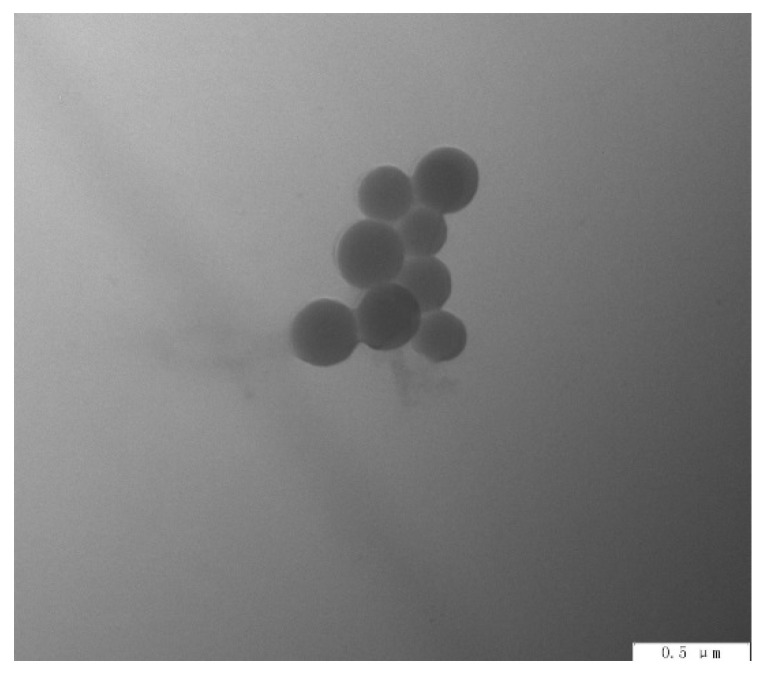
TEM morphology of N-2-HACC NPs.

As can be seen from Figure 9, N-2-HACC NPs prepared under optimized conditions had uniform particle size, good dispersion, and regular morphology.

**Figure 10:**
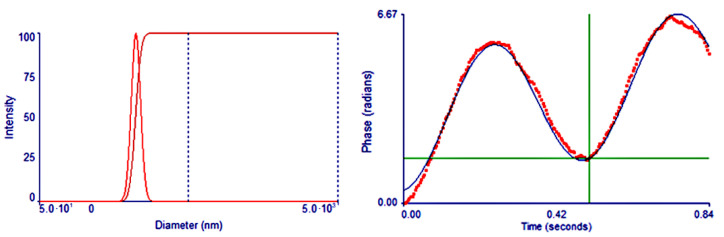
Zeta potential and size distribution of N-2-HACC NPs.

Average diameter of N-2-HACC NPs was 215.9 ± 44.0 nm, and zeta potential was + 32.31 ± 0.65 mV (Figure 10). As reported, the size of the nanoparticles affects the absorption of the nanoparticles by immune cells. Nanoparticles similar in size to pathogens (5–300 nm for viruses) are easily absorbed by antigen-presenting cells, which enhance the immune response [35]. In addition, zeta potential is an important influencing factor in mucosal drug delivery, while positively charged polymers are conducive to adsorbing negatively charged mucins, enhancing mucosal adhesion, and extending the drug residence time [36]. Therefore, the N-2-HACC NPs was expected to deliver drugs to the mucosa and achieve local administration.

### 3.3. N-2-HACC/PEDV Immune Effect

**Figure 11:**
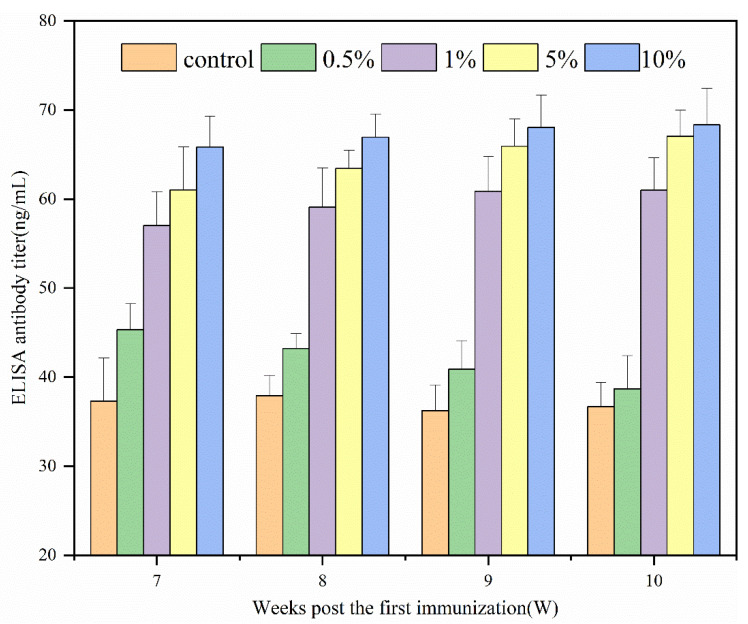
ELISA antibody dynamics regularity of serum in guinea pigs.

Figure 11 shows the immune effects of N-2-HACC/PEDV inactivated vaccines at varying adjuvant dosages. Since the prepared N-2-HACC/PEDV (10^7.0^ TCID_50_/mL) was a pink liquid, it was easily separated from the bottle wall by gentle shaking. From the above results, we can see that the ELISA antibody titer of each adjuvant group was higher than that of the control group. Except for the 0.5% (*w*/*v*) adjuvant group, the ELISA antibody titer of each adjuvant group was significantly different to the control group. After 10 weeks of immunization, each adjuvant group reached their maximum, with the antibody titer of the 10% (*w*/*v*) adjuvant group found to be the highest, followed by the 5% (*w*/*v*) adjuvant group. In contrast, the antibody titer of the two groups remained at a high level. Compared with previous reports, the N-2-HACC/PEDV inactivated vaccines prepared by us had less PEDV (1%) mixed in proportion, and the immune effect and immune time were better, indicating that the N-2-HACC/PEDV inactivated vaccines is an effective PEDV candidate vaccine [37].

## 4. Conclusions

The effectiveness of existing PEDV vaccine is still an outstanding problem. Chitosan and its derivative nanoparticles have demonstrated massive vaccine adjuvant potential, which makes them one of the current hotspots of research. In this study, the N-2-HACC showed excellent solubility and the capability to reduce the degree of substitution. In addition, it simplified the process of N-2-HACC synthesis and reduced the number of reagents used. As for the nanoparticles composed of N-2-HACC and β-GC, they were constructed by means of self-assembly and cross-linking. According to the relevant reports, 50–500 nm nanoparticles can be effectively absorbed by cells. The nanoparticles produced in this study demonstrated uniformity in particle size and high dispersibility, with the particle size falling below 500 nm. According to the immune research results, only a small number of nanoparticles and simple hybrid methods are required, and N-2-HACC/PEDV nanoparticles performed significantly in immunization and maintained a high level of antibody titer in the 10th week. Unsurprisingly, N-2-HACC self-assembled nanoparticles have attracted much attention as a safe and effective platform.

## Figures and Tables

**Table 1:** The vaccine composition with different adjuvant doses.

Group	N-2-HACC NPs (%, *w*/*v*)	PEDV (mL)	N-2-HACC NPs (g/mL)	Adjuvant Dose (mL)
1	0.5	1	0.01	1
2	1	1	0.02	1
3	5	1	0.1	1
4	10	1	0.2	1

**Table 2:** Experimental data of CS.

*C* (g/L)	t (s)	*η_r_*	ln*η_r_*	ln*η_r_/C*	*η_sp_*	*η_sp_/C*
-	92	-	-	-	-	-
6	474	5.1522	1.6394	0.2732	4.1522	0.6920
4	364	3.9565	1.3754	0.3438	2.9565	0.7391
2.4	204	2.2174	0.7963	0.3318	1.2174	0.5073
1.71	166	1.8043	0.5902	0.3451	0.8043	0.4704
1.33	147	1.5978	0.4686	0.3524	0.5978	0.4495

**Table 3:** Experimental data of N-2-HACC.

*C* (g/L)	t (s)	*η_r_*	ln*η_r_*	ln*η_r_/C*	*η_sp_*	*η_sp_/C*
-	92	-	-	-	-	-
5	178	1.9348	0.6600	0.1320	0.9348	0.1870
3.33	146.3	1.5092	0.4639	0.1393	0.5902	0.1773
2	123	13370	0.2904	0.1452	0.3370	0.1685
1.43	114	1.2391	0.2144	0.1499	0.2391	0.1672
1.1	108	1.1739	0.1603	0.1458	0.1739	0.1581

**Table 4:** Solubility of CS and N-2-HACC in different solvents at room temperature.

Sample Solvent	CS	N-2-HACC
	Solubility (mg/mL)	
Normal saline, 0.9% (*w*/*v*)	-	63.9 ± 0.71
Isopropyl alcohol	-	-
Deionized water	-	73 ± 1.41
Acetic acid, 1% (*w/v*)	35.1 ± 2.12	188.9 ± 4.95
Sodium hydroxide, 1% (*w/v*)	-	54.1 ± 0.99

“-” means insoluble.

## Data Availability

The raw/processed data required to reproduce these findings cannot be shared at this time as the data also forms part of an ongoing study.

## Supplementary Materials

The following are available online at https://www.mdpi.com/article/10.3390/polym13234097/s1, Figure S1: The Zeta potential of N-2-HACC; Figure S2: Relationship between C-lnηr/C and C-ηsp/C.
