# Supplementary figures and images for: Self-Assembly of Soluble Chitosan Derivatives Nanoparticles for Vaccine: Synthesis, Characterization and Evaluation
Source: Polymers (Basel). 2021 Nov 25;13(23):4097. doi: 10.3390/polym13234097 (PMC8659217; doi:10.3390/polym13234097)

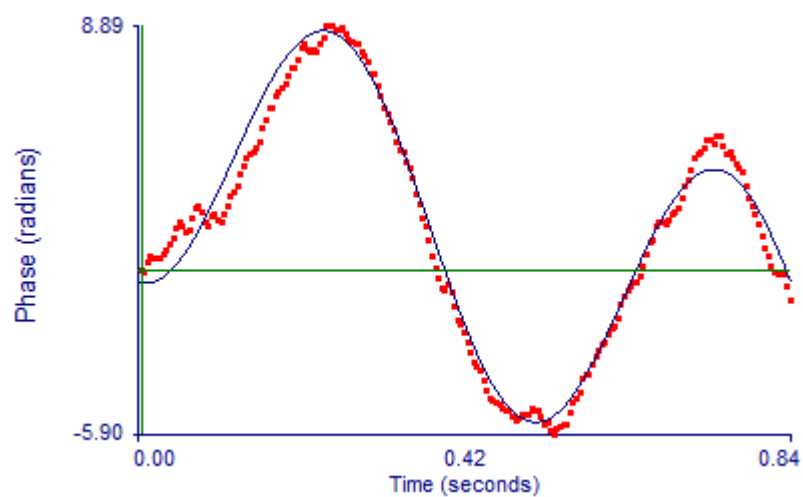

**Figure S1.** The Zeta potential of N-2-HACC.

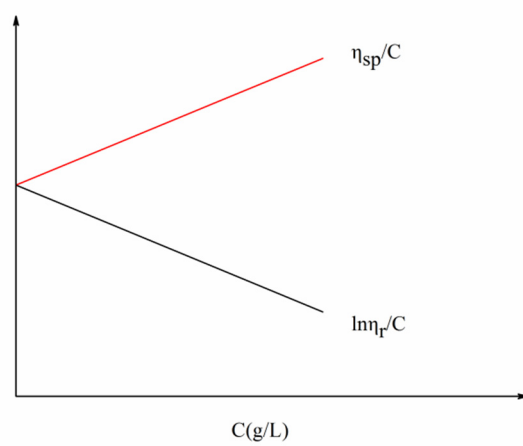

**Figure S2.** Relationship between  $C-\ln\eta_r/C$  and  $C-\eta_{sp}/C$ .

Supplement: Supplementary file 1 [file polymers-13-04097-s001.zip › polymers-1434119-supplementary.pdf]
